# Loss of E2F1 Extends Survival and Accelerates Oral Tumor Growth in HPV-Positive Mice

**DOI:** 10.3390/cancers7040895

**Published:** 2015-12-08

**Authors:** Rong Zhong, John Bechill, Michael T. Spiotto

**Affiliations:** Department of Radiation and Cellular Oncology, The University of Chicago, 900 E. 57th Street, Chicago, IL 60637, USA; rzhong@uchicago.edu (R.Z.); jbechill@uchicago.edu (J.B.)

**Keywords:** human papillomavirus 16, E2F1 transcription factor, genes, tumor suppressor, mice, transgenic, lethal

## Abstract

The Human Papillomavirus (HPV) is associated with several human cancers, including head and neck squamous cell carcinomas (HNSCCs). HPV expresses the viral oncogene E7 that binds to the retinoblastoma protein (RB1) in order to activate the E2F pathway. RB1 can mediate contradictory pathways—cell growth and cell death via E2F family members. Here, we assessed the extent to which E2F1 mediates lethality of HPV oncogenes. Ubiquitous expression of the HPV oncogenes E6 and E7 caused lethality in mice that was associated with focal necrosis in hepatocytes and pancreatic tissues. Furthermore, all organs expressing HPV oncogenes displayed up-regulation of several E2F1 target genes. The E2F1 pathway mediated lethality in HPV-positive mice because deletion of *E2F1* increased survival of mice ubiquitously expressing HPV oncogenes. E2F1 similarly functioned as a tumor suppressor in HPV-positive oral tumors as tumors grew faster with homozygous loss of *E2F1* compared to tumors with heterozygous loss of E2F1. Re-expression of E2F1 caused decreased clonogenicity in HPV-positive cancer cells. Our results indicate that HPV oncogenes activated the E2F1 pathway to cause lethality in normal mice and to suppress oral tumor growth. These results suggest that selective modulation of the E2F1 pathway, which is activated in HPV tumors, may facilitate tumor regression.

## 1. Introduction

The Human Papillomavirus is associated with many epithelial cancers, including those afflicting the oropharynx and anogenital regions [1,2,3]. HPV enters the skin through microabrasions in order to infect stem cells in the basal epithelial layer [4]. The viral oncogenes of HPV, namely E7, promote DNA synthesis in the maturing epithelial layer in order to facilitate viral replication [4,5]. To facilitate proliferation in differentiated cells, E6 and E7 target canonical tumor suppressor pathways including the proteins TP53 and the retinoblastoma protein (RB1), respectively. The loss of RB1 and other pocket protein family members, including RBL1 (p107) and RBL2 (p130), enable cells to progress through the cell cycle and to initiate DNA synthesis. Typically, abnormal activation of the cell cycle also initiates cell death pathways that safeguard against malignant transformation. E6, by targeting p53 and other anti-apoptotic pathways, inhibits these cell death pathways, thereby predisposing abnormally-proliferating cells to malignant transformation. Consequently, the genes used to promote viral replication inadvertently predispose a fraction of HPV-infected individuals to develop dysplastic and, subsequently, malignant epithelial lesions. However, virally-infected cells also express the HPV gene E2 that regulates E6 and E7 expression and viral replication in order to promote viral maturation [6]. The malignant transition of HPV-infected cells to cancer coincides with the integration of the HPV viral genome that disrupts E2 expression resulting in E6 and E7 overexpression and the activation and/or inhibition of downstream oncogenic and tumor suppressor pathways, respectively.

Paradoxically, the RB-E2F1 pathway has been implicated both in control on proliferation and cell death. Heterozygous loss of *RB1* makes mice prone to develop thyroid and pituitary tumors [7,8,9]. By contrast, homozygous loss of *RB1* results in embryonic lethality due to abnormal proliferation, apoptosis, and cellular differentiation [9,10,11]. Embryonic lethality is associated with abnormal erythroid and neuronal differentiation [11]. This dichotomous effect of *RB1* loss is mediated, in part, by E2F family members that regulate the transcription of genes involved in the cell proliferation and cell death [12]. In particular, E2F1 promotes both cellular proliferation, as well as p53-dependent and p53-independent cell death [13,14,15,16,17]. E2F1 promotes cellular proliferation pathways as loss of E2F1 inhibits tumor development in tumor prone mice heterozygous for *RB1* [18]. By contrast, E2F1 also promotes cell death pathways as loss of E2F1 diminishes apoptosis and delays embryonic lethality in mice with homozygous RB1 loss [19]. Therefore, RB1 mediates paradoxical cell proliferation and cell death signals, in part, via E2F1.

Previous reports have not described the lethality of HPV oncogenes in transgenic mice consistent with the innocuous HPV infections in humans. The majority of these HPV transgenic mouse models used constitutively active tissue-specific promoters including cytokeratin 14 and cytokeratin 10 that limited E6 and/or E7 expression to the skin which may better tolerate oncogene expression [20,21,22]. However, few, if any, reports have addressed the extent to which the expression of HPV E6 and E7 oncogenes in other tissues negatively impacts cell survival. Given that *RB1* loss caused embryonic lethality, we hypothesized that HPV oncogenes may also cause lethality and inhibit tumor growth via an E7-RB1-E2F pathway [23].

Here, we used a tamoxifen (TAM)-inducible HPV transgenic mouse model [24] to ubiquitously express the HPV oncogenes E6 and E7 in developing embryos and in adult mice. TAM treatment induced HPV oncogene expression in several tissues and caused lethality in adult mice within 60 days. Tissues from mice ubiquitously expressing E6 and E7 demonstrated necrosis and increased transcription of E2F1 target genes. Genetic ablation of *E2F1* rescued HPV-induced lethality in adult mice. Furthermore, loss of *E2F1* increased HPV-positive oral tumor growth that was associated with increased tumor cell proliferation. Together, our results indicated that the E2F1 pathway, which is controlled by the HPV E7 oncogene, inhibits cell proliferation causing lethality in HPV-positive mice and suppressing HPV-positive oral tumor growth.

## 2. Results and Discussion

### 2.1. Results

#### 2.1.1. Tissue Specific HPV Oncogene Expression Causes Embryonic Lethality

Homozygous loss of *RB1* causes embryonic lethality [9,10,11]. Since the HPV oncogene E7 targets the RB1, we tested if ubiquitous expression of HPV oncogenes also results in embryonic lethality. We used a recently generated transgenic mouse, iHPV mice, that contains a Cre-regulated LoxP-Stop-LoxP-iE6E7-IRES-Luciferase (LSL-E6E7) transgene [24]. Activation of Cre recombinase excises the inhibitory LSL sequence in order to express E6 and E7 under a ubiquitous CMV enhancer/chicken β-actin promoter/rabbit b-globin slice acceptor (CAG) synthetic promoter. To test the extent to which HPV oncogenes cause embryonic lethality, we bred female iHPV mice homozygous for the LSL-E6E7 transgene to male mice heterozygous for a CMV-Cre transgene that ubiquitously expresses Cre in all tissues. While all offspring contained the iE6E7 transgene, only four of 42 mice (9.5%) contained the CMV-Cre transgene. By contrast, breeding CMV-Cre mice to control FVB mice resulted in 55 of 100 mice having the CMV-Cre transgene (55.0%; *p* < 0.001; Table 1), consistent with the expected frequency of the heterozygous allele. Since the presence of both CMV-Cre × iHPV transgenes lead to ubiquitous E6 and E7 oncogene expression and resulted in fewer offspring than predicted, our data suggested that HPV oncogene expression resulted in embryonic lethality when expressed in all organs.

**Table 1:** Tissue-specific expression of HPV oncogenes causes embryonic lethality.

Parents	Total Mice	Number of Litters	Age of Analysis (Days)	iHPV	Cre	*p Value Cre*
CMV-Cre × iHPV ^1^	42	8	12.6 ± 3.7	42 (100.0%)	4 (9.5%)	<0.0001
CMV-Cre × FVB	100	10	14.0 ± 1.7	0 (0.0%)	55 (55.0%)	
Nestin-Cre × iHPV ^2^	42	8	12.6 ± 1.7	42 (100.0%)	0 (0.0%)	<0.0001
Nestin-Cre × FVB	28	3	14.5 ± 2.2	0 (0.0%)	18 (64.3%)	
Albumin-Cre × iHPV ^3^	55	6	13.3 ± 1.3	55 (100.0%)	26 (47.3%)	N.D.
GFAP-Cre × iHPV ^3^	34	4	9.3 ± 1.3	34 (100.0%)	18 (52.9%)	N.D.
K14-Cre × iHPV ^4^	49	5	11.4 ± 3.7	49 (100.0%)	49 (100.0%)	N.D.
PDX1-Cre × iHPV ^3^	34	6	13.0 ± 2.5	34 (100.0%)	19 (55.9%)	N.D.

^1^ CMV-Cre transgenic mice and iHPV mice were hemizygous and homozygous for the respective transgene and, therefore, 50% of offspring should contain Cre recombinase and 100% of offspring should contain iHPV oncogene; ^2^ Nestin-Cre transgenic mice were hemizygous the Cre transgene and, therefore, 50% of offspring should contain Cre recombinase and 100% of offspring should contain iHPV oncogene; ^3^ Albumin-Cre, GFAP-Cre and PDX1-Cre transgenic mice were hemizygous for the respective transgene and, therefore, 50% of offspring should contain Cre recombinase and 100% of offspring should contain iHPV oncogene; ^4^ K14-Cre transgenic mice were homozygous the Cre transgene and, therefore, 100% of offspring should contain Cre recombinase and 100% of offspring should contain iHPV oncogene.

Given that HPV infections are not associated overt skin toxicity [4,5], the embryonic lethality of CMV-Cre × iHPV mice suggested that different tissues may have distinct tolerances to HPV oncogene expression. Therefore, we used transgenic mice expressing Cre recombinase under different tissue-specific promoters to assess tolerance of different tissues to E6 and E7 oncogene expression. To induce tissue specific expression of E6 and E7, we bred iHPV mice to: (1) Nestin-Cre mice to express E6 and E7 predominantly in the central and peripheral nervous system; (2) Albumin-Cre mice to express E6 and E7 predominantly in the liver; (3) GFAP-Cre mice to express E6 and E7 predominantly in astrocytes; (4) K14-Cre mice to express E6 and E7 predominantly in the skin; and (5) PDX1-Cre mice to express E6 and E7 predominantly in the pancreas. Breeding Nestin-Cre mice to iHPV mice resulted in no double-transgenic mice in 42 offspring tested while breeding Nestin-Cre mice to control FVB mice to resulted in 18 of 28 offspring having Cre transgene (64.3%; *p* < 0.001; Table 1). Therefore, HPV oncogene expression in Nestin^+^ tissues caused embryonic lethality. By contrast, breeding iHPV mice to Albumin-Cre mice, GFAP-Cre mice, K14-Cre mice, or PDX1-Cre mice resulted in double-transgenic mice at expected Mendelian ratios. Therefore, different tissues have distinct sensitivity to HPV oncogenes resulting embryonic lethality when expressed ubiquitously or in Nestin^+^ tissues.

#### 2.1.2. Ubiquitous Expression of HPV Oncogenes Caused Tissue Necrosis and Lethality in Adult Mice

**Figure 1:**
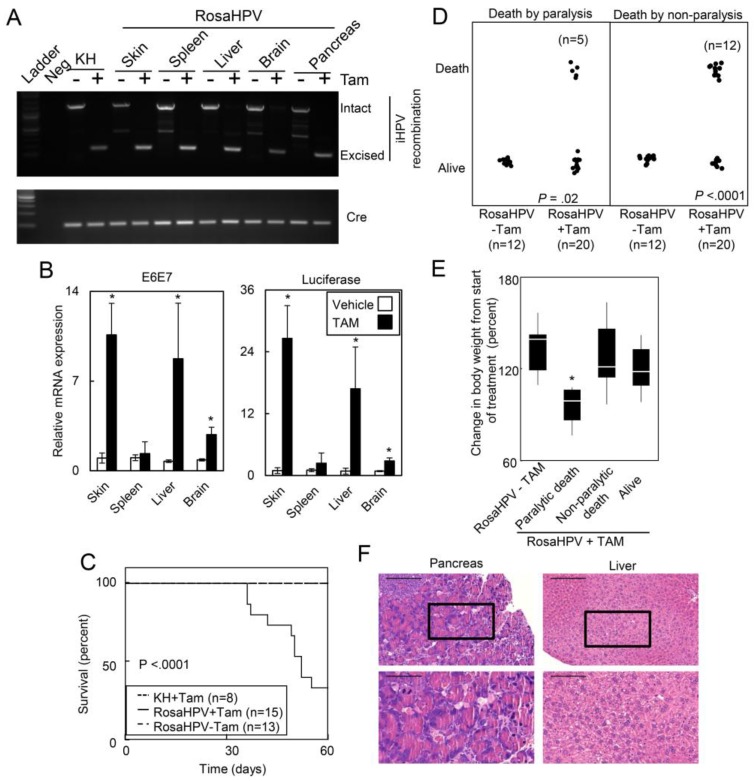
Ubiquitous induction of HPV oncogenes caused lethality in adult mice. (**A**) TAM-induced recombination of the iHPV transgene in the skin, spleen, liver, brain and pancreas of RosaHPV mice. PCR identification of LSL-E6E7 transgene recombination (upper panel) or the control Cre transgene; (**B**) TAM-induced expression of E6E7 and luciferase transgenes in RosaHPV mice treated with TAM or vehicle for 5d. qRT-PCR for E6E7 and luciferase transgenes in RosaHPV mice treated with or without TAM. Data represent from organs isolated from five individual mice per group. Error bars represent standard deviation (st. dev.) *p* values determined by Student’s *t*-test; (**C**) TAM treatment caused lethality in RosaHPV mice but not in control mice. RosaHPV mice or KH mice were treated with TAM or vehicle. Mice were monitored for survival and depicted in a Kaplan-Meier survival plot. *p* value determined by Log-rank test; (**D**) TAM treatment caused death associated with or without paralysis. Of 17 RosaHPV mice that died, five displayed evidence of paralysis while 12 had normal limb movement. Paralysis was scored as positive if mice had impaired gait or loss of lower limb function. *p* value determined by Likelihood Ratio; (**E**) paralyzed RosaHPV mice that died had significant weight loss. Weights of RosaHPV mice treated with vehicle or TAM were measured at the start of the experiment and twice weekly. Change in body weight was calculated using body weight at the time of death or end of the experiment compared to body weight at the start of the experiment. Data displayed in quantile box-and-whisker plots where upper and lower whiskers represent the first and fourth quartiles, respectively, and the upper and lower boxes represent the second and third quartiles, respectively. *p* value determined by t-test with appropriate Bonferroni corrections; and (**F**) liver and pancreas demonstrated necrosis. Histology of indicated organs Rosa-HPV mice treated with TAM. Upper panels scale bar = 500 μM; Lower panels scale bar = 100 μM. Box in lower magnification field area depicted in the higher magnification field. * denotes *p* < 0.01.

Since ubiquitous expression of HPV caused embryonic lethality, we assessed the extent to which expression of HPV oncogenes caused death in adult mice. To overcome embryonic lethality with constitutive HPV oncogene expression, we bred iHPV mice to Rosa-CreER^tam^ mice (RosaHPV) that expressed a TAM-regulated Cre recombinase driven by a ubiquitous Rosa promoter. Tamoxifen treatment of RosaHPV mice caused recombination of the LSL-E6E7 transgenes in the skin, spleen, liver, brain and pancreas (Figure 1A). Recombination of the LSL-E6E7 recombination was associated with the induced expression of the E6E7 transgene as well as an internal ribosomal entry site-linked luciferase transgene (Figure 1B). Within 60 days after TAM treatment, only 33.3% of RosaHPV mice survived (five of 15 mice) while no deaths were observed in vehicle treated Rosa-HPV mice or TAM-treated K14-CreER^tam^-iHPV mice in which HPV oncogenes were induced in the skin (*p* < 0.0001; Figure 1C). Of note, five of 20 TAM-treated RosaHPV mice experienced paralysis in their hind limbs that was associated with significant weight loss (Figure 1D,E). TAM-treated Rosa-HPV mice demonstrated necrosis in the pancreas and liver (Figure 1F). However, no obvious malignant lesions were observed on necropsy. Therefore, ubiquitous expression of HPV oncogenes caused lethality in adult mice associated with tissue necrosis but not overt tumor development.

**Figure 2:**
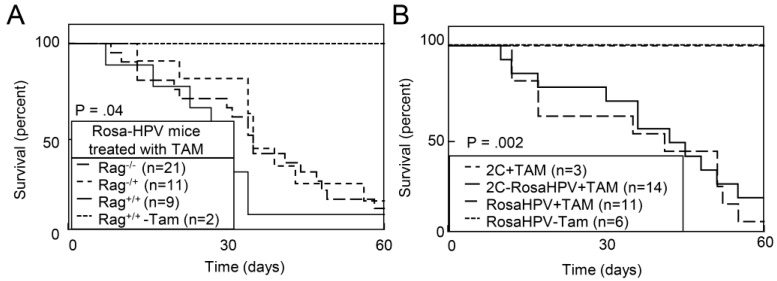
TAM treatment of RosaHPV mice did not cause lethality due to immune responses against induced HPV oncogenes. (**A**) TAM treatment caused lethality in immunodeficient Rosa-HPV-Rag1^−/−^ mice. RosaHPV mice also containing the Rag1^−/−^, Rag1^+/−^ or Rag1^+/+^ genotypes were treated with TAM. In addition, control RosaHPV-Rag1^+/+^ mice were treated with vehicle (-TAM); (**B**) TAM caused lethality in immunosuppressed RosaHPV mice also transgenic for the 2C T cell receptor. RosaHPV mice containing the 2C TCR transgene contain >95% of T cells specific for the 2C T cell receptor making mice immunosuppressed and unable to reject immunogenic tissues. *p* value determined by log-rank test.

Since Rosa-HPV mice induced expression of the HPV oncogenes E6 and E7, as well as a luciferase reporter, we excluded the possibility that these induced proteins served as neoantigens stimulating lethal autoimmune responses. We bred Rosa-HPV mice to Rag1^−/−^ mice in order to generate T cell- and B cell-deficient mice (RosaHPV-Rag1^−/−^). In addition, we bred Rosa-HPV mice to a 2C TCR transgenic mice [25] where greater than 90% of T cells are specific against an α-Ketoglutarate dehydrogenase self-antigen (RosaHPV-2C) resulting in a functionally immunodeficient state [26]. TAM treatment caused similar decreases in survival for RosaHPV-Rag1^+/+^, RosaHPV-Rag1^+/−^ and RosaHPV-Rag1^−/−^ mice (Figure 2A). Similarly, TAM treatment caused similar decreases in survival for RosaHPV and RosaHPV-2C mice (Figure 2B). Therefore, the decreased survival caused by HPV oncogene expression was not explained by immune responses generated against induced antigens.

#### 2.1.3. HPV Oncogenes Induce Expression of E2F1 Target Genes in the Skin, Spleen, Liver and Brain

**Figure 3:**
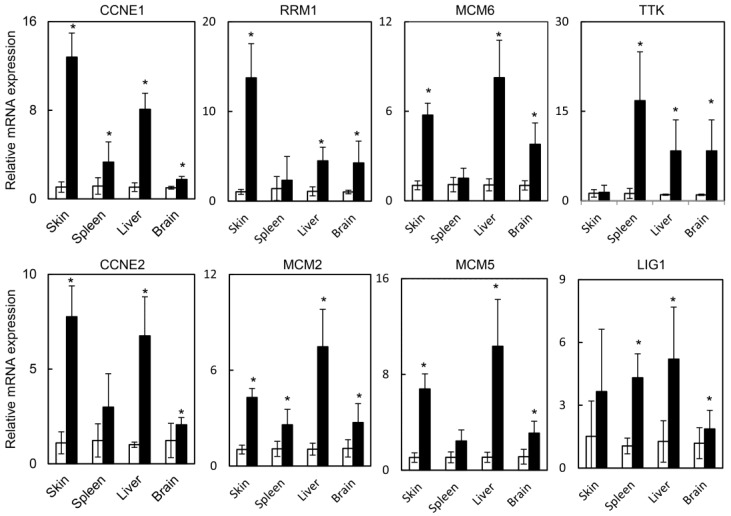
E2F target genes are induced in different organs of RosaHPV mice treated with TAM. The skin, spleen, liver, and brain from RosaHPV mice treated with TAM (filled bars) or vehicle (open bars) subjected to qRT-PCR for the E2F target genes *CCNE1*, *RRM1*, *MCM6*, *TTK*, *CCNE2*, *MCM2*, *MCM5*, and *LIG1*. Data from tissues isolated from five mice per group and assayed in duplicate. * denotes *p* < 0.01. Error bars represent SD. *p* values determined by Student’s *t*-test.

Previous groups demonstrated that loss of *RB1* resulted in embryonic lethality that was partially mediated by E2F1 [19]. HPV oncogenes may also cause embryonic lethality by targeting RB1 to induce E2F1 activity. Therefore, we assessed the extent to which HPV oncogene expression induced E2F target genes in several normal tissues. Using the skin, spleen, liver, and brain isolated from RosaHPV mice treated with TAM or vehicle, we assayed the expression E2F1 target genes cyclin E1 and E2 (*CCNE1* and *CCNE2*), minichromosome maintenance complex component 2, 5, and 6 (*MCM2*, *MCM5*, and *MCM6*), ribonucleotide reductase M1 (*RRM1*) and ttk protein kinase (*TTK*). TAM treatment caused induction of E2F1 target genes in the skin, spleen, liver, and brain (Figure 3). Notably, *RRM1* was induced predominantly in the skin while *TTK* was induced predominantly expressed in the spleen, liver, and brain. Overall, these data indicated that HPV oncogene expression up-regulated E2F1 target genes consistent with the activation of the E2F pathway.

#### 2.1.4. Deletion of *E2F1* Rescues HPV Oncogene Lethality in Adult Mice

**Figure 4:**
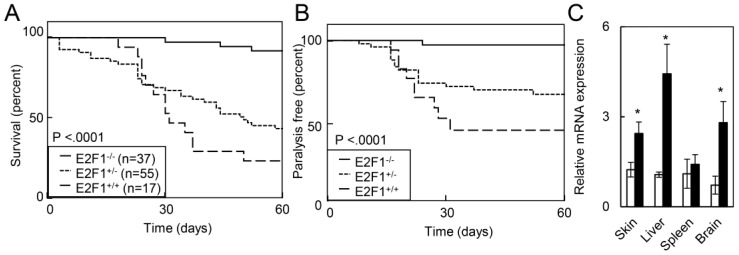
Loss of E2F1 rescued HPV oncogene lethality and paralysis in adult mice. (**A**) E2F1 loss rescued HPV oncogene lethality in adult RosaHPV mice. RosaHPV-E2F1^+/+^ (long dashed line), RosaHPV-E2F1^+/−^ (short dashed line), and RosaHPV-E2F1^−/−^ (solid line) mice were treated with TAM and monitored for survival; (**B**) Rosa-HPV-E2F1^+/+^, Rosa-HPV-E2F1^+/−^ and Rosa-HPV-E2F1^−/−^ mice were treated with TAM and monitored for paralysis. *p* value determined by Log-rank test; (**C**) E2F1 expression increased in the skin, spleen, and brain after TAM. RosaHPV mice were treated with vehicle (open bars) or TAM (filled bars) and, 5d later, E2F1 mRNA expression was detected by qRT-PCR.

Loss of *E2F1* has been previously shown to extend the survival of *RB1* deficient embryos from embryonic day 13 to embryonic day 17.5 [19]. Given that HPV oncogenes induced expression of E2F1 target genes, we assessed how *E2F1* loss impacted the survival of RosaHPV mice after TAM treatment. Compared to RosaHPV mice with intact or heterozygous loss of *E2F1*, homozygous loss of *E2F1* increased the median survival of RosaHPV mice after TAM treatment (Figure 4A). (Median survival: RosaHPV-E2F1^−/−^: not reached; RosaHPV-E2F1^+/−^: 49 days; RosaHPV-E2F1^+/+^: 30 days; *p* < 0.0001.) Furthermore, fewer RosaHPV-E2F1^−/−^ mice displayed gait abnormalities or paralysis compared to RosaHPV-E2F1^+/−^ mice or RosaHPV-E2F1^+/+^ mice (*p* < 0.0001; Figure 4B). To determine if E2F1 dependent lethality was determined by tissue-specific differences in *E2F1* gene expression, we assessed *E2F1* gene expression in the skin, spleen, liver, and brain in RosaHPV mice treated with vehicle or TAM. We observed induction of *E2F1* gene expression after TAM treatment in the skin, spleen and brain suggesting that tissue-specific lethality of HPV oncogene expression is unlikely to be due to differences in *E2F1* gene induction. Therefore, E2F1 mediates lethality caused by HPV oncogenes in adult mice.

#### 2.1.5. *E2F1* Inhibits HPV-Positive Tumor Growth and Clonogenicity

**Figure 5:**
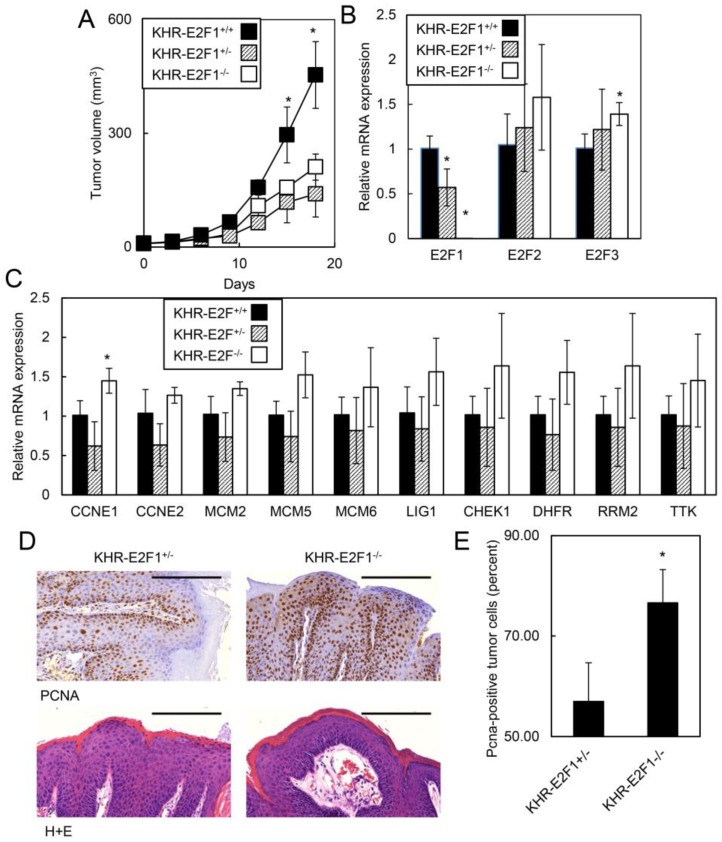
*E2F1* suppressed HPV oral tumor growth. (**A**) HPV oral tumors grew faster with loss of E2F1. KHR-E2F1^+/+^(*n* = 3), KHR-E2F1^+/−^ (*n* = 4) and KHR-E2F1^−/−^ (*n* = 5) mice were treated with TAM to induce primary oral tumors and tumor growth was monitored every 3 d. Representative of three independent experiments; (**B**) KHR-E2F1^−/−^ mice expressed low levels of *E2F1* transcripts but similar or higher levels of *E2F2* and *E2F3* transcripts. qRT-PCR analysis of *E2F1*, *E2F2*, and *E2F3* was performed on RNA isolated from KHR-E2F1^+/+^, KHR-E2F1^+/−^, and KHR-E2F1^−/−^ oral tumors; (**C**) loss of *E2F1* caused similar or higher levels of E2F1 target genes. RNA isolated from KHR-E2F1^+/+^, KHR-E2F1^+/−^ and KHR-E2F1^−/−^ oral tumors was assessed for expression of the indicated genes using qRT-PCR. Average of three tumors performed in duplicate in Figure 5B,C; (**D**) loss of *E2F1* increased tumor cell proliferation. KHR-E2F1^+/^^−^ and KHR-E2F1^−/−^ oral tumors were assessed for PCNA by immunohistochemistry; and (**E**) quantitation of PCNA in KHR tumors with intact or deficient *E2F1*. Eight random fields from four individual KHR-E2F1^+/−^ and KHR-E2F1^−/−^ oral tumors were assessed for PCNA nuclear staining as previously described [27]. * denotes *p* < 0.01. Error bars represent SD. *p* values determined by Student’s *t*-test.

**Figure 6:**
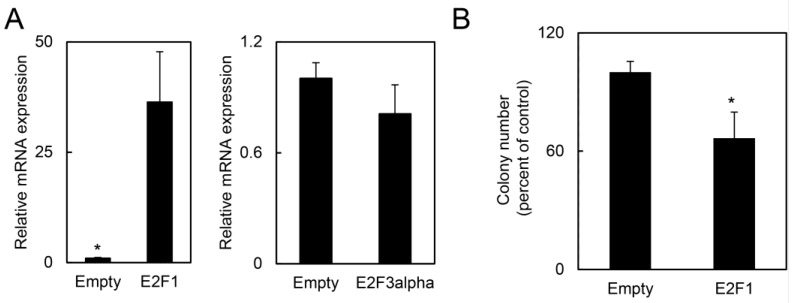
E2F1 overexpression suppressed clonogenicity in HPV-positive cells. (**A**) Compared to control transfected cells, HeLa cells transfected with E2F1 vector expressed elevated levels of *E2F1* but similar levels of *E2F3a*. Total RNA was extracted from HeLa cells transfected with E2F1 or control vectors and subjected to qRT-PCR for the indicated genes; and (**B**) overexpression of E2F1 causes loss of clonogenicity in HPV-positive HeLa cells. HeLa cells were transfected with vectors expressing E2F1 or control enhanced green fluorescence protein and assessed for clonogenic survival. * denotes *p* < 0.01. Error bars represent SD. *p* values determined by Student’s *t*-test.

Since HPV oncogenes caused lethality in adult mice that was dependent on *E2F1*, we hypothesized that *E2F1* may also inhibit the growth of HPV-positive tumors. Previously, we developed an HPV oral tumor model, KHR mice, containing the K14-CreER^tam^ transgene, the LSL-iE6E7 transgene and a LSL-Kras^G12D^ transgene [24]. TAM treatment activated CreER^tam^ in epithelial cells to induce E6E7 oncogenes as well as the mutant Kras^G12D^ oncogene resulting in oral tumor formation. KHR mice with homozygous loss of *E2F1* developed oral tumors that grew faster than KHR mice with heterozygous loss of *E2F1* (Tumor volume at d18: 453.7 mm^3^ for KHR-E2F1^−/−^ *vs.* 139.7 mm^3^ for KHR-E2F1^+/−^; *p* = 0.0004; Figure 5A). Although KHR-E2F1^−/−^ mice had no *E2F1* expression as assessed by qRT-PCR (Figure 5B), E2F target genes were expressed at similar or even higher levels for *CCNE1* compared to KHR-E2F1^+/+^ or KHR-E2F1^+/−^ mice with intact E2F1 (Figure 5C). Given that the expression of other E2F family members were not affected by *E2F1* deletion, our results suggest that E2F2, E2F3, and other E2F transcription factors may compensated for *E2F1* loss to maintain proliferation stimuli in HPV-positive cells. While all tumors displayed invasive features, KHR-E2F1^−/−^ oral tumors had increased proliferating cells as assessed by PCNA immunohistochemistry (Figure 5D,E). Since *E2F1* inhibited human HPV-positive oral tumor growth, we assessed if increased *E2F1* expression also inhibited HPV-positive cancer cell growth. HPV-positive HeLa cells transfected with *E2F1* or control expression vectors demonstrated increased *E2F1* expression but not *E2F3* (Figure 6A). HeLa cells expressing *E2F1* had decreased clonogenicity compared to HeLa cells expressing the control vector (Figure 6B). Thus, our data indicates that E2F1 is a target for HPV oncogenes that mediates conflicting tumor suppressive pathways in HPV-positive oral tumors.

### 2.2. Discussion

Here, we show that HPV oncogenes activated the E2F1 pathway to cause normal tissue lethality and to inhibit tumor growth. Ubiquitous expression of HPV oncogenes resulted in embryonic and adult lethality likely by causing cell death as evidenced by necrosis in the pancreas and liver. The lethality of HPV oncogenes was unlikely due to immune responses against the induced oncogenes because immunodeficient HPV-positive mice still died. Furthermore, this lethality was unlikely due to tumor development due to oncogene expression as we did not observe any evidence for neoplastic transformation in solid tissues or in the blood. The lethality of HPV oncogenes were most likely mediated through the E7-RB1-E2F1 pathway as abrogation of the downstream *RB1* target, *E2F1*, improved survival in adult mice. Analogous to the lethality caused by HPV oncogenes, HPV oncogenes also activated tumor suppressive pathways in oral tumors as evidence by faster tumor growth rates with *E2F1* loss and by the inhibition of clonogenicity in cells overexpressing E2F1. Since the suppression of tumorigenesis was apparent only with *E2F1* loss, the cumulative effect of HPV oncogene expression still resulted in accelerated oral tumor growth. Nevertheless, these observations suggest HPV oncogenes can activate tumor suppressor pathways that may ultimately be manipulated for therapeutic gain.

To show that *E2F1* caused lethality and inhibited tumor growth in cells expressing HPV oncogenes, we used mice transgenic for E6 and E7 as well as HPV-positive cancer cell lines. Transgenic promoters may express HPV oncogenes at levels well above the expression of E6 and E7 by endogenous viral promoters in HPV-infected cells. Consequently, we cannot exclude that our transgenic models induced E2F1 activation at substantially higher levels that would be seen in normal tissues or tumorigenic keratinocytes expression viral oncogenes at physiological levels. Furthermore, we also drew a conclusion using the HPV-positive HeLa cancer cell line that has additional chromosomal abnormalities and impaired cellular pathways that may not reflect the effects of biologically relevant HPV oncogene expression on cell proliferation and lethality.

We observed that ubiquitous expression of HPV oncogenes leads to tissue necrosis and lethality in adult mice. Previously, Eichten *et al.* and Jones *et al.* have shown that expression of the HPV oncogene E7 led to cell death and apoptosis in diploid fibroblasts via inhibition of RB1 control on activation of the E2F1 pathway, due to E7-RB1 binding [28,29]. Similarly, loss of the E7 target, *RB1*, resulted in embryonic lethality [9,10,11]. While these previous experiments avoided E7 induced cell death via expression of E6, we find that HPV lethality may occur even in mice expressing both E6 and E7. Since the loss of *RB1* activates p53-dependent and p53-indepement cell death pathways [17], our findings are consistent with the model that E7 controls E2F1 which mediates growth-suppressive signals via p53-independent pathways in the setting of HPV E6 oncogene expression. Furthermore, distinct tissues may have different tolerances to HPV oncogenes. To this end, we observed embryonic lethality when that HPV oncogenes were expressed in Nestin-positive tissues, while mice expressing HPV oncogenes regulated by other tissue specific promoters were viable. Since E2F1-target genes such as *CCNE1* were differentially expressed in the brain compared to other target tissues, it is interesting to speculate that tissue-specific differences in E2F1 transcriptional targets may mediate tissue necrosis and may explain the central nervous system dysfunction associated with HPV oncogene expression. Therefore, our results indicate that HPV oncogenes, such as E7, caused organ toxicities that were likely associated with distinct organ tolerances.

Our results also find that HPV oncogenes caused lethality in adult mice and suppressed oral tumor growth via the E2F1 pathway. Field *et al.* demonstrated that homozygous loss of *E2F1* both stimulated proliferation as well as decreased cell death leading to spontaneous tumor development [30]. Similarly, we observed increased proliferation in HPV oral tumors with *E2F1* loss. By contrast, overexpression of *E2F1* in mouse skin lead to hyperproliferation and apoptosis that required the loss of p53 for tumor development [31]. Although transgenic expression of other E2F family members, including *E2F3α*, also induced apoptosis in the skin [32], *E2F1* is likely the central mediator of cell death, as apoptosis induced by *E2F3a* required *E2F1* [33]. Similarly, *E2F1* expression caused apoptosis in the neural tissues even though *E2F1*, *E2F2*, and *E2F3* contributed to cell proliferation in the brain [34]. Although we only observed necrosis in the liver and pancreas, we did not observe increased apoptosis which may be due to low levels of apoptosis in specific organs that occurred over several weeks. Nevertheless, our data support the observations that HPV oncogenes caused lethality in normal mice and suppressed tumor growth via E2F1.

E2F1 may mediate contradictory signals to both promote and to inhibit tumor growth depending on the level of RB1 inactivation and on distinct post-translational modifications. E2F1 may promote tumor growth when the E2F1 pathway is partially active and E2F1 may suppress tumor growth when the E2F1 pathway is overactive. Yamasaki *et al.* demonstrated that E2F1 promoted tumor growth as mice with *RB1* haploinsufficiency and, consequently, partial E2F pathway activation formed fewer tumors with homozygous *E2F1* deletion [18]. Similarly, HPV oncogenes may, in part, rely on E2F1 to facilitate cell proliferation as we observed that hemizygous loss of *E2F1* caused slower HPV tumor growth compared to tumors with intact *E2F1*. By contrast, complete loss of *RB1* caused increased activation of the E2F1 pathway resulting in embryonic lethality that was partially reversed by *E2F1* deletion [19]. Similarly, we observed that HPV oral tumors grew faster with complete loss of *E2F1* consistent with the growth inhibitory role of E2F1 in tissues with complete loss of *RB1*. As an alternative explanation of the contradictory growth signals, Zheng *et al.* has proposed that distinct post-translational patterns of arginine methylation in E2F1 governs the cell growth or cell death signal [35]. While expression of different *E2F1* arginine mutations did not further impact clonogenicity of HPV cells in preliminary experiments, it is possible to post-translational changes govern the role of E2F1 in HPV-positive tumors. Thus, multiple mechanisms may enable E2F1 both to promote and to inhibit HPV tumor growth.

Consequently, E2F1 may serve as a potential therapeutic target for human head and neck cancers. Targeting E2F1 may include altering arginine methylation patterns, inducing E2F1 expression or targeting E2F1 specific cell death pathways. Since HPV-induced cancers depend on expression of E7 resulting in E2F1 activation [36], specific enhancement of apoptotic pathways mediated by E2F1 may serve as effective approach against an essential pathway in HPV-positive cancers. In addition, given the distinct sensitivity of different tissues to HPV oncogenes, further investigation into how tissue specific pathways increase sensitivity to HPV oncogenes may identify other anti-neoplastic targets dependent or independent of E2F1 activation. Furthermore, E2F1 may also mediate tumor suppressive functions in other cancers, including breast cancer [37] and such anti-cancer therapies targeting E2F1 may also be useful in HPV-negative tumors that have homozygous deletion of *RB1* or over-activation of MYC that also mediates apoptosis via E2F1 [38]. Therefore, E2F1 may serve as a therapeutic target for both HPV-positive and HPV-negative cancers.

## 3. Experimental Section

### 3.1. Animals

All mice were maintained under specific pathogen-free conditions and used according to protocols approved by The University of Chicago (Chicago, IL, USA) Institutional Animal Care and Use Committee and Institutional Biosafety Committee (Protocol ACUP 72136). iHPV mice containing the Lox-Stop-Lox-HPV-Luc (LSL-E6E7) transgene [24] and KH mice expressing the LSL-E6E7 and K14-CreER^tam^ transgene [39] have been described. Rosa-CreER^tam^ mice (B6;129-*Gt(ROSA)26Sor^tm1(cre/ERT)Nat^*/J) [40], K14-Cre mice (B6N.Cg-Tg(KRT14-cre)1Amc/J) [41], Albumin-Cre mice (B6N.Cg-Tg(Alb-cre)21Mgn/J) [42], PDX1-Cre mice (B6.FVB-Tg(Pdx1-cre)6Tuv/J) [43], CMV-Cre mice (B6.C-Tg(CMV-cre)1Cgn/J) [44], Nestin-Cre mice (B6.Cg(SJL)-TgN(NesCre)1Kln) [45] and GFAP-Cre mice (FVB-Tg(GFAP-cre)25Mes/J) [46] were obtained from Jackson Laboratories (Bar Harbor, ME, USA). iHPV mice on an FVB background were bred to Albumin-Cre mice, PDX1-Cre mice, CMV-Cre mice, Nestin-Cre mice on a C57BL/6 congenic background to generate mice with tissue-specific recombination of the LSL-E6E7 transgene on a (C57BL/6 × FVB)F1 background. iHPV mice and KH mice were backcrossed onto a C57BL/6 background as previously described and iHPV mice were bred with Rosa-CreERtam on a C57BL/6 background.

### 3.2. Reagents and Antibodies

Tamoxifen was purchased from Sigma (St. Louis, MO, USA). DMBA and TPA were dissolved in acetone. Tamoxifen was dissolved in sunflower seed oil at 20 mg/mL and 0.1 mL was injected intraperitoneally daily for five days. All oligonucleotides were synthesized by IDT.

### 3.3. HPV Recombination

All PCR reactions were performed using 2× Go Taq Green Master Mix (Promega, Madison, WI, USA). For detection of LSL-E6E7 recombination, gDNA was isolated and the LSL-HPV transgene was amplified by PCR using the primers: forward primer: 5ʹ-CAACGTGGTTATTGTGCTG-3ʹ corresponding to the 3ʹ end of the CAG promoter and the reverse primer: 5ʹ-GGTAACTTTCTGGGTCGCTCCT-3ʹ corresponding to the 5ʹ region of the E6E7 gene. The 1629 bp amplicon represents the unexcised HPV vector and the 388 bp amplicon represents the excised iE6E7 gene.

### 3.4. Quantitative RT-PCR

RNA was extracted from the indicated organs of Rosa-HPV mice 5d after treatment with TAM or vehicle or from KHR, KHR-E2F1^+/−^ and KHR-E2F1^−/−^ oral tumors at the end of the experiment. Total RNA was isolated using TRIzol (Invitrogen, Carlsbad, CA, USA), and treated with DNase I. The cDNA was generated using ProtoScript^®^ First Strand cDNA Synthesis Kit (NEB, Ipswich, MA, USA) and subjected to qPCR using SYBR Green Supermix (Biorad, Hercules, CA, USA). The endogenous GAPDH (Glyceraldehyde 3-phosphate dehydrogenase) gene was used as a reference gene. QPCR Primers: E6E7 primers (E6E7-F: 5ʹ-ATTTGCAACCAGAGACAACT and E6E7-R:5ʹ-ACAGGTCTTCCAAAGTACGA), Luc primers (Luc-F: 5ʹ-ACTGGGACGAAGACGAACAC and Luc- R: 5ʹ-GGCGACGTAATCCACGATCT), and GAPDH mouse primers (GAPDH-F: 5ʹ-AATGTGTCCGTCGTGGATCT and GAPDH-R: 5ʹGGTCCTCAGTGTAGCCCAAG). In addition, validated mouse primer pairs for E2F1, E2F2, E2F3, LIG1, Ttk6, MCM2, MCM5 MCM6, CCNE1, CCNE2, RRM2, and B2M were obtained from PrimePCR^TM^ Assays (Bio-Rad, Hercules, CA, USA). QPCR was carried out using iQ™ SYBR^®^ Green Supermix with hot-start iTaq DNA polymerase and iQ™ 5 Multicolor Real-time QPCR Detection System Machine (Bio-rad). CT values for individual samples were subtracted from the corresponding CT values for GAPDH or B2m (∆∆CT). Normalized fold expression was obtained using the –log2^∆∆^^CT^ formula.

### 3.5. Colony Formation Assay

The HPV-positive cell line HeLa was obtained from Kenneth Alexander (The University of Chicago) and was validated by IDEXX laboratories nine-loci STR testing. Cells were maintained in complete DMEM (cDMEM) with 10% fetal bovine serum plus L-glutamine and Penn/Strep at 37 °C. HeLa cells were plated in six-well plates and transfected with a bicistronic expression plasmid containing human E2F-1 and EGFP (EX-F0047-Lv215, Genecopiea, Rockville, MD, USA) or the empty plasmid containing EGFP using lipofectamine 2000. After 24 h, a 1000 cells were split into six-well plates and incubated for 13 days. After 13 days, the media was removed and washed with PBS. The cells were then fixed with methanol for 5 min and then stained with 1% crystal violet for 30 min. Colonies were counted and the experiment was performed in triplicate.

## 4. Conclusions

Here, we find that HPV oncogenes caused lethality *in utero* and in adult mice that is associated with necrosis in normal tissues without the development of neoplastic lesions. HPV oncogene lethality was mediated, in part, by the HPV oncogene E7 targeting E2F1, as loss of E2F1 reversed lethality in adult mice. Furthermore, loss of E2F1 potentiated HPV-positive oral tumor growth and overexpression of E2F1 decreased the clonogenicity of HPV-positive cancer cells. Understanding how to manipulate cell death pathways activated by HPV oncogenes via E2F1 may serve as novel approaches to treat head and neck cancers and other cancers in general.
